# Supplementary material for: Pressure induced superconductivity in MnSe
Source: Nat Commun. 2021 Sep 14;12:5436. doi: 10.1038/s41467-021-25721-1 (PMC8440596; doi:10.1038/s41467-021-25721-1)
Supplement: Supplementary file 1 — Supplementary Information [file 41467_2021_25721_MOESM1_ESM.pdf]

Supplementary materials

Supplementary Note 1-Equivalence compression pressure

Based on the refined lattice parameters of the Mn(Se-S) system [30], we estimated the equivalent compression pressure (E.C.P) in MnSe by systematic sulfur substitution, using the third-order Birch-Murnaghan equation of state ($P = 3f(1+2f)^{5/2}K_0(1-3/2f(4-4K'))$), where the Eulerian finite strain is given by $f = 1/2((V/V_0)^{-2/3}-1)$; K_0 = 72 GPa; K' = 4.3 GPa reported by Catherine McCammon [31]. The results suggest the E.C.P. of MnS (relative to MnSe) is ~13.2 GPa as shown in Table S1.

Supplementary Table 1. Lattice constant, cell volume, V/V_0 ratio, Eulerian finite strain (f) and equivalence compression pressure (E.C.P.) data for $MnSe_{1-x}S_x$ system

	Lattice constant (Å)	volume (Å ^{3})	V/V_0	f	E.C.P. (GPa)
x = 0	5.454	162.235	1	0	0
x = 0.05	5.441	161.078	0.993	0.002	0.523
x = 0.25	5.4	157.464	0.971	0.01	2.292
x = 0.5	5.34	152.273	0.939	0.022	5.23
x = 0.75	5.269	146.28	0.902	0.034	9.319
x = 1	5.212	141.584	0.873	0.048	13.151

Supplementary Note 2-Image of the electrical contacts arrangement in a DAC

High pressure resistivity measurements used a DAC with 400 μm culets. A rhenium gasket was covered by cubic-BN powders for insulating the electrical leads in a sample chamber filled with hexagonal-BN as pressure transmitting medium. Gold foils were used for preparing electrode leads to connect sample and gold wires. Fig. S1 shows the detail of the contacts arrangement.

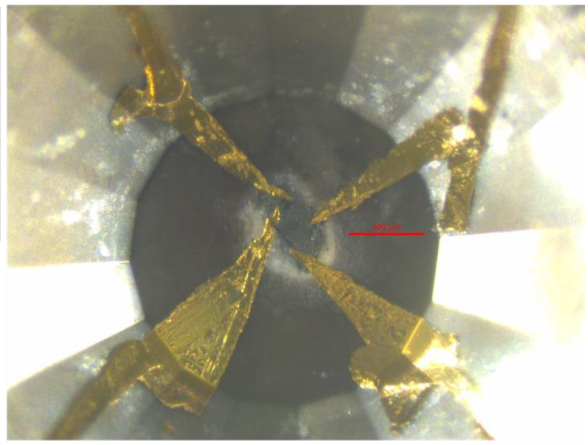

Supplementary Fig. 1. The optical image of the electrical contacts arrangement in DAC .

Supplementary Note 3-The definition of onset T_C in RT measurements.

The onset Tc of R(T) measurements is defined as the intersection temperature of two adjacent fitting lines of dρ/dT as shown in Supplementary Fig. 2., here we use three pressure points where are 16, 23, and 42 GPa as an example.

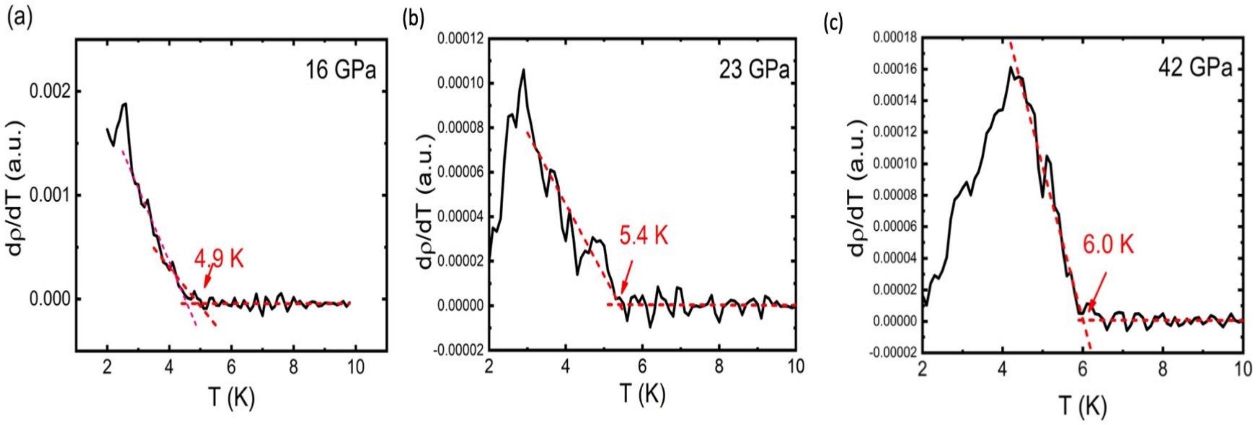

Supplementary Fig. 2. The Tc is estimated bu the curve of dρ/dT versus T at (a) 16 GPa, (b) 23 GPa, (c) 42 GPa.

Supplementary Note 4-The magnetic susceptibility at low pressure.

For magnetic susceptibility measurements, a mini-DAC fabricated from BeCu alloy, which was adapted into a Quantum Design Magnetic Property Measurement System (MPMS), was used for ultrasensitive magnetization measurements under high pressures. A pair of 300-μm-diameter culet-sized diamond anvils was used. The gaskets were made from nonmagnetic Ni–Cr–Al alloy. Each gasket was pre-indented to ∼20 μm in thickness, and a ~120-μm-diameter hole was drilled to serve as the sample chamber. The mixture of methanol and ethanol in a ratio of 4:1 was used as the pressure transmitting medium. A piston-cylinder-type high-pressure cell, compatible with MPMS, was used when performing low pressure measurements up to 1.3 GPa, where the

pressure medium was Daphne-7373 oil and the pressure manometer was a lead piece. Supplementary Fig. 3 displays the χ vs. T and d χ/dT vs. T at pressure up to 1.2 GPa. Figure S4 shows χ vs. T during unloading process. During unloading, our results showed that T_N was more reversible than the T_s (Supplementary Fig. 5). After fully releasing the pressure, by comparing the result with zero pressure data before applying pressure, we found out $\Delta T_N \sim 15$ K while $\Delta T_s \sim 30$ K. We would like to point out during unloading a third anomaly appeared between 240 K and 280 K (Supplementary Fig. 4). The peak position of this anomaly decreases as pressure decreases. Supplementary Fig. 6-1 presents how T_c at different pressures are determined by dM/dT vs. T. Supplementary Fig. 6-2 shows the T_c at 51. 64 GPa under different H up to 2500 Oe determined by two approaches.

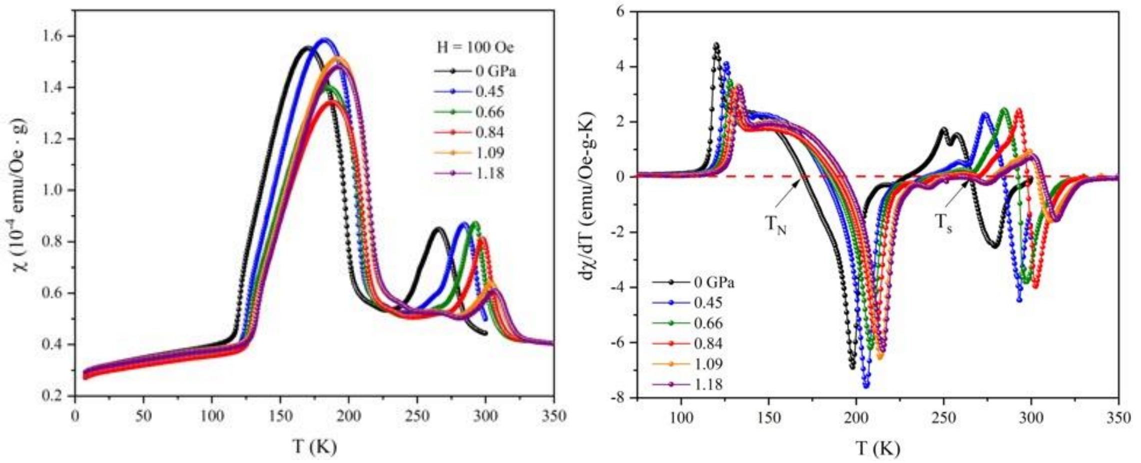

Supplementary Fig. 3. χ vs. T and d χ/dT vs. T under pressure up to 1.2 GPa.

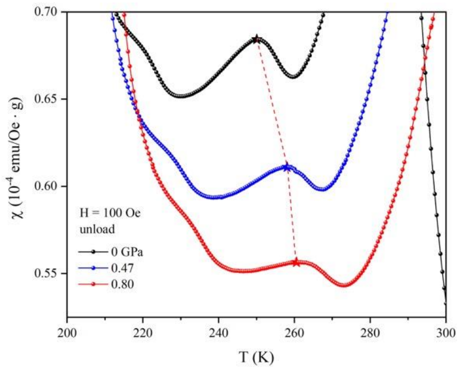

Supplementary Fig. 4. χ vs. T during unloading process.

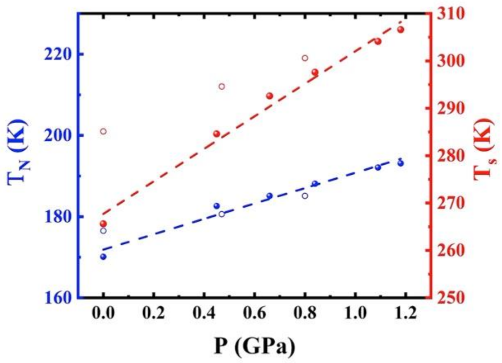

Supplementary Fig. 5. T_N vs. P and T_s vs. P under pressures up to 1.2 GPa. Solid symbols represent

results during loading process while open symbols show data collected during unloading process.

Supplementary Note 5-The definition of onset T_C in MT measurements.

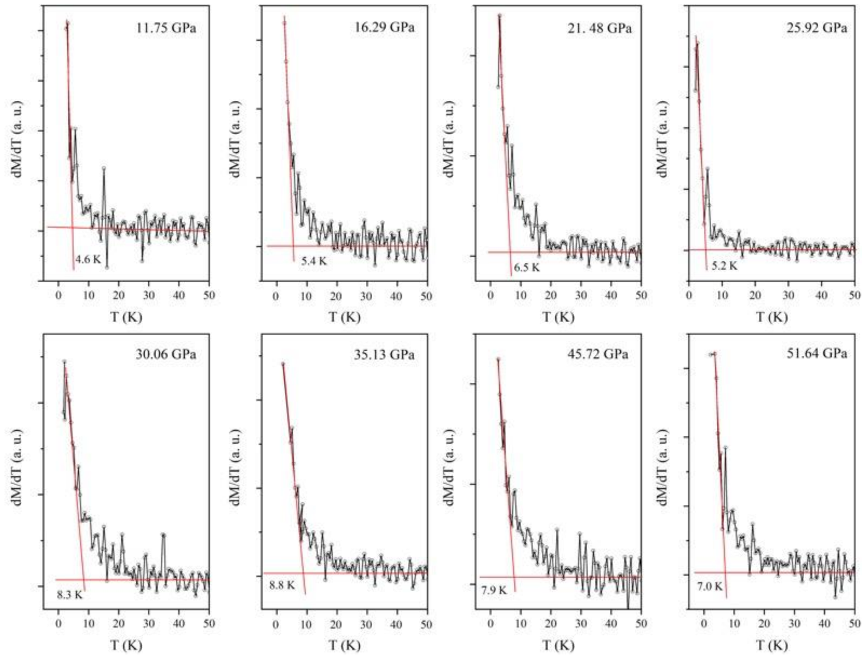

Supplementary Fig. 6-1. T_c at different pressures determined by dM/dT vs. T.

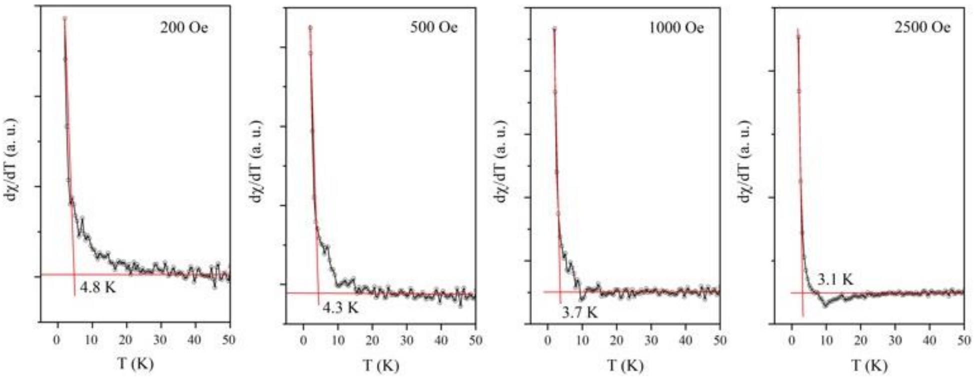

Supplementary Fig. 6-2. T_c at 51 GPa under different H up to 2500 Oe by dM/dT vs. T.

Supplementary Note 6- The ruby spectra at different pressures.

Supplementary Fig. 7 shows the ruby spectra at different pressures for R(T) measurements.

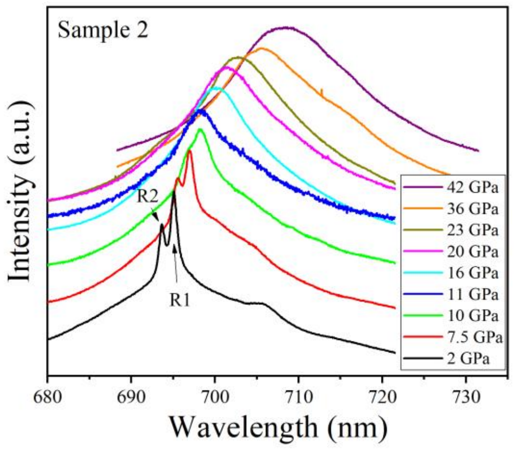

Supplementary Fig. 7 Pressure dependent of ruby fluorescence spectra for R(T).

Supplementary Note 7- In-situ XRD for liquid pressure medium case.

We perform the pressure dependent XRD with liquid pressure medium (methanol and ethanol in a ratio of 4:1). The representative diffraction patterns (9.5、11 and 16 GPa) are collected in Supplementary Fig. 8(1). The peak positions in these patterns are marked the miller index. The analysis of 11 GPa pattern is shown in Supplementary Fig. 8(2). For comparison, we plot volume change under pressure for MnSe using gas (helium) and Liquid (M:E 4-1) as the pressure transmission medium (PTM). The results show that the appearance of orthorhombic phase at ~12 GPa. Secondly, the collapse of mix phase in MnSe to single orthorhombic phase appears at ~ 16GPa in liquid PTM, , which is much lower than that observed in helium gas PTM.

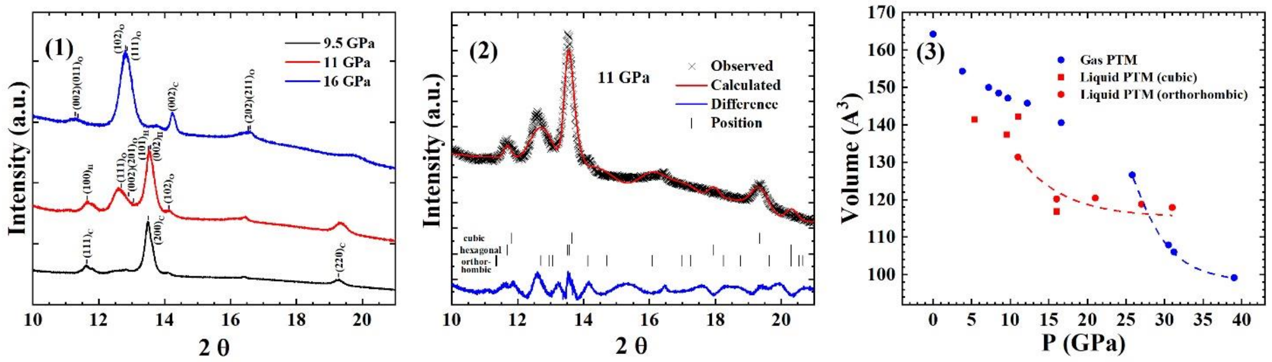

Supplementary Fig. 8 (1) X-ray diffraction pattern of MnSe using methanol and ethanol in a ratio of 4:1 as the pressure medium at 9.5, 11, and 16 GPa. (2) Typical Rietveld refinement of MnSe at 11 GPa. (3) The pressure dependence of volume for MnSe using gas (helium) and Liquid (M:E 4-1) as the pressure medium. The dash line in color to present the structure of MnSe in the collapse process to

orthorhombic phase.

Supplementary Note 8-The estimation of micro-strain

We used the following formula (FWHM * cos (θ)) ^{2} =(wavelength/d)^2 +(strain * sin(θ))^2 (θ : the diffraction angle, which is half of the center angle of diffraction peak in the I vs. 2 θ profile; d: the average grain size.) to estimate the variation of mean grain size and micro-strain of MnSe under pressure above that MnSe shows single orthorhombic phase. Supplementary Fig. **9-(1)** to Supplementary Fig. **9-(2)** are the fitting curve of each pattern obtained using helium gas as PTM, and the calculated results are listed in Supplementary Table 2. The mean size of the orthorhombic phase seems to remain about the same (though with small increase). And the calculated micro-strain generated under pressure at the single orthorhombic phase using gas PTM is in the order of 1%, but consistently (with our previous estimate) increases in the pressure range investigated.

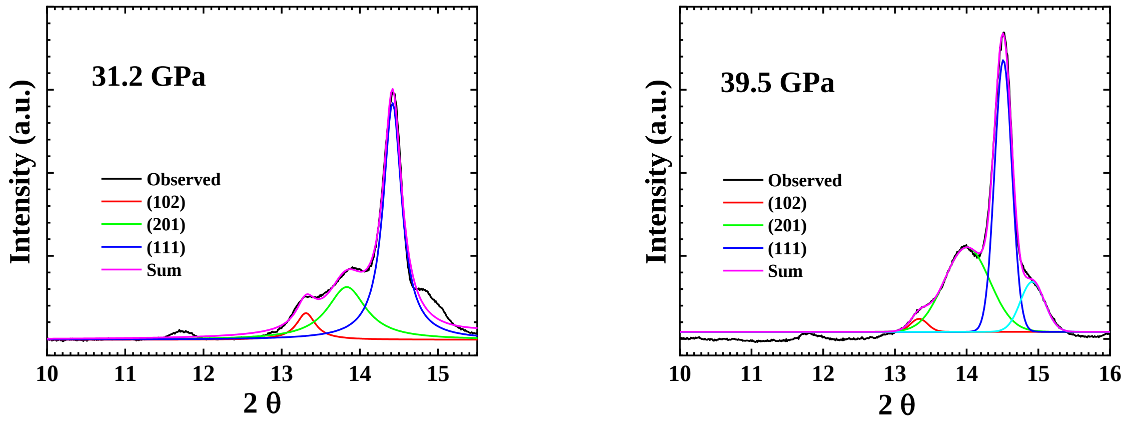

Supplementary Fig. **9.** Multi-peak fitting of MnSe with helium as the pressure medium at **(1)** 31.2, and **(2)**39.5 GPa.

	Peak	FWMH (deg.)	FWMH (rad.)	Peak center (deg.)	d (nm)	ε (%)	T_C^* (K)
31.2 GPa	(111)	0.285(4)	0.00497	14.418(1)	12.(7)	0.6(5)	5.85
	(102)	0.284(9)	0.00495	13.310(9)			
39.5 GPa	(111)	0.283(2)	0.00494	14.511(4)	14.(2)	1.5(8)	6.25
	(102)	0.279(9)	0.00487	13.337(9)			

Supplementary Table 2. List of FWHM (deg.), FWHM(rad.), peak center (deg.), and both grain size (d) and strain (ε) from (111) and (102) peak fitting result of orthorhombic phase with helium gas as the pressure medium. The corresponding Tc value as under same PTM condition are also listed in the same table for comparison. (*: The Tc value is extracted from the Fig.1-(f) by interpolation.)

Supplementary Note 9- The total energy in cubic、hexagonal and orthorhombic phase in MnSe.

We also performed first-principles calculations using Quantum Espresso with norm-conserving local-density approximation pseudopotential to estimate the total energy/formula unit in different phases. The results are shown in Supplementary Table 3. The cubic phase is most energetically favored at pressures $<$ 10 GPa, while the hexagonal (H) and orthorhombic (O) phases have about the same energy in the range of 10~30 GPa and it is only slightly lower than the cubic (C) phase. This is consistent with the phase diagram of Fig. 7 that C, H and O phases are all mixed between 12 ~30 GPa. Around P~ 40GPa, O phase has much lower energy than the other two phases.

Supplementary Table 3. Total energy/formula unit in meV. The lowest energy at each pressure is set to 0.

**P (GPa)**	**cubic**	**hexagonal**	**orthorhombic**
**0**	0	4.9	4.8
**10**	19.3	0	0.4
**20**	3.8	0.3	0
**30**	13.1	0.5	0
**40**	476.9	92.9	0
